# Ecophysiological study of green microalgae isolated from the grit crust of the Atacama Desert

**DOI:** 10.1111/jpy.70182

**Published:** 2026-06-05

**Authors:** K. Glaser, F. Kuschel, L. R. Prelle, P. Jung, U. Karsten

**Affiliations:** ^1^ Institute of Biological Sciences, Biology/Ecology Technical University Bergakademie Freiberg Freiberg Germany; ^2^ Institute for Biological Sciences, Applied Ecology and Phycology University Rostock Rostock Germany; ^3^ XCEL – Extreme Cryptogam Ecology Lab University of Applied Sciences Kaiserslautern Kaiserslautern Germany; ^4^ Interdisciplinary Faculty, Department of Maritime Systems University of Rostock Rostock Germany

**Keywords:** biological soil crust, desiccation, Klebsormidium, PI curve

## Abstract

Terrestrial algae play a critical, yet often overlooked, role in the functioning of biological soil crusts (biocrusts), which are considered microecosystems, particularly in extreme environments. This study investigated the ecophysiological traits of green algae isolated from the grit crust—a unique biocrust type reported a few years ago in the coastal Atacama Desert (National Park Pan de Azúcar), Chile. We assessed photosynthetic performance, temperature tolerance, and desiccation recovery in four strains: *Pseudochlorella signiensis* strain C11, *Lobosphaera incisa* strain LC2, and two strains of *Klebsormidium elegans*, C12 and C14. The results revealed significant interspecific variation, with *P. signiensis* exhibiting the highest photosynthetic capacity and both *Klebsormidium* strains demonstrating efficient light utilization. Desiccation recovery at very low relative air humidity was limited across all species, potentially reflecting the influence of frequent fog events in the coastal region of the Atacama, which result in a moderate air humidity. This study fills a critical knowledge gap regarding terrestrial algal ecology in South America and highlights the physiological traits enabling algal survival in one of the world's most extreme environments, providing insights into the resilience of biocrust communities in the face of environmental change.

**Abbreviations**
3N BBM: Bold basal medium with triple nitrogen
*l*_c_
: light compensation point
*l*_k_
: light saturation
MAT: mean annual temperatures
NPQ: non‐photochemical quenching
PAM: pulse amplitude modulation chlorophyll fluorometer
P‐I curve: photosynthetic‐irradiance curve
P_max_
: maximum photosynthetic rate
Y(II): yield of photosystem II

## INTRODUCTION

Biological soil crusts (biocrusts) are complex biotic assemblages comprising bacteria, algae, fungi, lichens, and mosses, which establish intimate associations with soil particles at the soil‐atmosphere interface. Through the biologically induced aggregation of soil particles, these communities form a cohesive, living surface layer. Unlike physical or chemical crusts, biological soil crusts are fundamentally reliant on the diverse metabolic activities and capabilities of their living organisms (Belnap et al., 2001). They provide more ecological niches for the organisms compared to barren soil. The abundance of microorganisms is higher compared to in soil and thus, more biotic interactions with a cross‐phylum character are possible (Glaser et al., 2022; Kurth et al., 2023). Further, the biocrusts grow on soil, and some organisms are able to vertically move into deeper layers with lower radiation intensity and less desiccation; for example, soil diatoms can glide along particles and the Cyanobacterium *Microcoleus sociatus* moves within its sheath made of extracellular polymeric substances (Bondoc‐Naumovitz et al., 2025; Garcia‐Pichel et al., 1996). Biocrusts predominantly colonize semiarid and arid ecosystems, which collectively constitute over 30% of the Earth's terrestrial surface, and estimates suggest that biocrusts constitute more than 12% of the global living ground cover (Rodriguez‐Caballero et al., 2018). However, they are also present in temperate, boreal, and Arctic biomes where microenvironmental conditions permit their establishment (Corbin & Thiet, 2020; Gall et al., 2022; Kern et al., 2019).

Biocrusts predominantly occupy terrestrial ecosystems that lack or exhibit only sparse coverage by vascular plants (Belnap et al., 2001). Here, they exert profound influences on the structure and function of the inhabiting drylands by fulfilling numerous ecological roles. Biocrusts are known to enhance the stability of highly erodible substrates and soils and modulate local hydrological cycles, a function of particular significance in regions characterized by limited precipitation (Belnap et al., 2001). In addition, biocrusts contribute to edaphic fertility by mediating biogeochemical nutrient‐cycling processes. A particularly large number of biocrust‐associated cyanobacteria are capable of fixing atmospheric nitrogen and contributing to bioweathering processes that lead to the dissolution of phosphorus‐rich minerals (Concostrina‐Zubiri et al., 2022; Elbert et al., 2012). In addition to nitrogen and phosphorus sequestration, biocrusts provide organic carbon to the ecosystem via photosynthetic carbon fixation, thereby supporting trophic interactions within the local food web. This function is particularly relevant in nutrient‐deficient environments, in which biocrusts make up the dominant lifeform (Belnap et al., 2001; Elbert et al., 2012).

The grit crust were only recently described as a specific type of biocrusts (Jung et al., 2020). Biocrusts can be distinguished into various types, based on color, morphology, and species composition (Weber et al., 2022). Jung et al. (2020) observed a new type of biocrust in the National Park Pan de Azúcar of the Atacama Desert, which is known as one of the driest deserts on Earth, hence providing rather precarious conditions for life. However, the arid coastal belt of the Atacama Desert is regularly influenced by fog events, which are sufficient to cause regular wet–dry cycles, thereby supporting biological activity (Jung et al., 2020). In this region, the authors described the hitherto unknown, highly unique grit crust as a new biocrust type. This community was dominated by lichens of the family Caliciaceae encrusting the lower surface of quartz and granitoid stones (approximately <6 mm), which hosted a diverse set of green algal photobionts of the genus *Trebouxia* (Jung et al., 2024). In addition, free‐living cyanobacteria, green algae, and fungi contribute to the community, which was recently investigated based on an isolation approach (Jung et al., 2025). The grit crust community covers a significant portion of the coastal Atacama Desert (350 km^2^ with coverages between 20% and 80%), providing ecological services such as protection of the soil from sporadically occurring splash erosion; contribution to the accumulation of soil carbon, nitrogen, and phosphorus; and facilitation of soil formation by bioweathering (Jung et al., 2020).

Very little data exist on biocrusts and biocrust‐associated microalgae from Chile or even from South America in general. The underlying lack of studies was noticed by Büdel et al. (2016), who mentioned in their review only one algal and 40 Cyanobacterial species from South American biocrust communities. Forest and Weston (1966), Patzelt et al. (2014), and, more recently, Jung et al. (2025) reported exclusively on the diversity of cyanobacteria from arid locations in northern Chile; reports on biocrusts in the Caatinga, Brazil (semiarid tropical vegetation), were published only a few years ago (Szyja et al., 2019). Samolov et al. (2019, 2020) were the first to address the biodiversity of microalgae and cyanobacteria in biocrusts sampled along a climatic gradient from south to northern Chile using a polyphasic approach, but they did not sample the grit crust in the National Park Pan de Azúcar of the Atacama Desert.

For the present investigation, we studied various preliminary characterized green algae (Chlorophyta, Streptophyta) isolated from the grit crust (Jung et al., 2025) and evaluated their ecophysiological traits in terms of light requirements for photosynthesis and temperature, and their desiccation tolerance. We investigated two coccoid Chlorophyta species (*Pseudochlorella signiensis* C11 and *Lobosphaera incisa* LC2) and two Streptophyta isolates of *Klebsormidium elegans*, which are all known to be closely associated with different types of biocrusts (Büdel et al., 2016; Jung et al., 2022; Mikhailyuk et al., 2019; Samolov et al., 2019). We hypothesized that grit‐crust‐associated green algae from the Atacama Desert exhibit broad ecological tolerances in terms of desiccation and temperature and have a high physiological plasticity in terms of light requirements for efficient photosynthesis due to the dynamic conditions, the fog‐driven wet/dry cycles, and the extreme temperature fluctuation in coastal regions of the Atacama Desert. Our study aimed to present ecophysiological data derived from single organisms from the cross‐phylum biocenosis of the grit crust in order to supplement data gained from the full biocrust community presented in, for example, Jung et al. (2020).

## MATERIALS AND METHODS

### Strains and culture conditions

**FIGURE 1:**
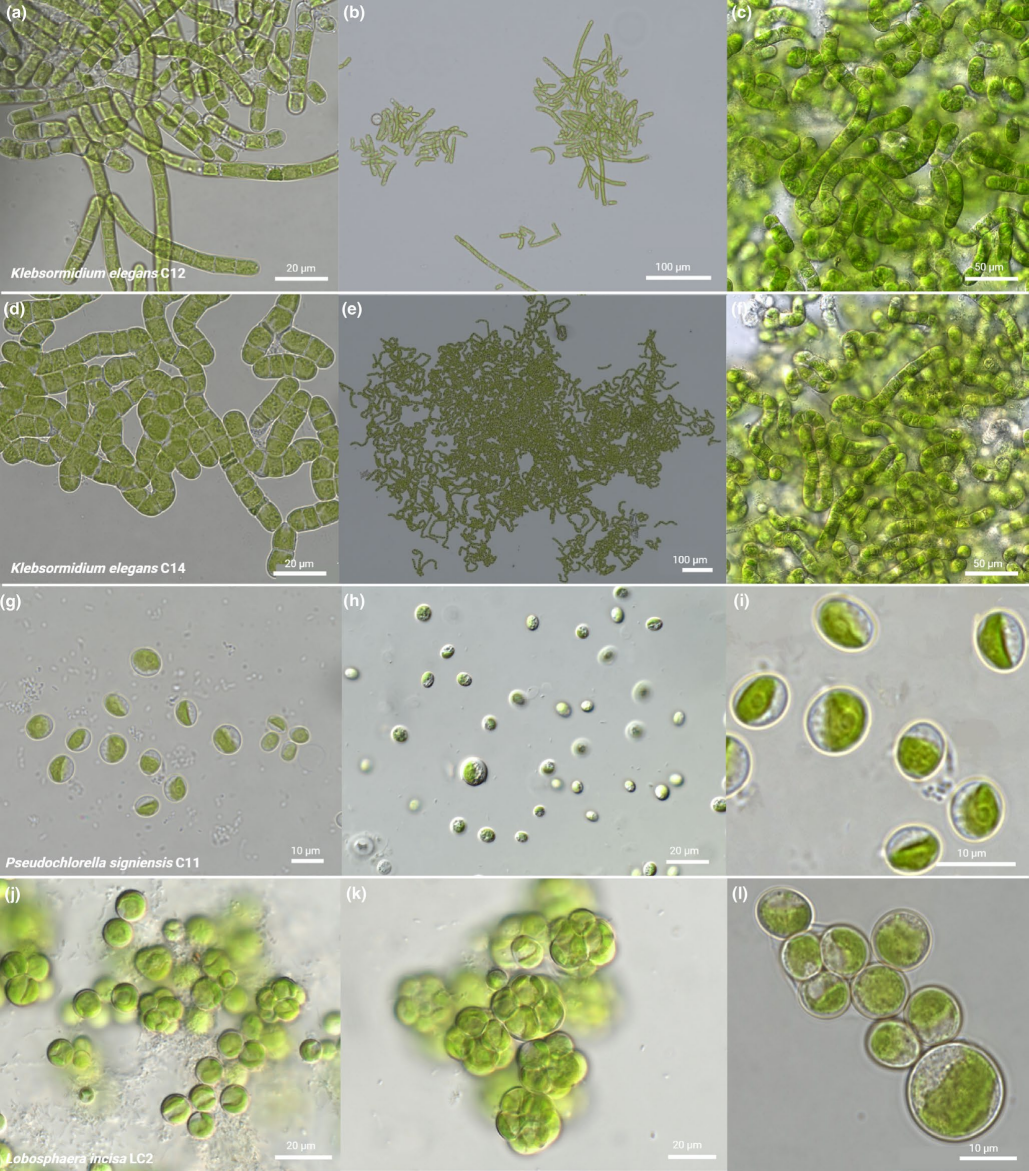
Micrographs of the isolated green algae. (a–c) *Klebsormidium elegans* C12, (d, e) *Klebsormidium elegans* C14, (g–i) *Pseudochlorella signiensis* C11, (j–l) *Lobosphaera incisa* LC2.

For this study, four green algal strains were used, which were previously isolated in 2024 from the grit crust of the National Park Pan de Azúcar in the Atacama Desert of Chile (Jung et al., 2025). Based on morphological traits and preliminary molecular–taxonomical data (18S and SSU rRNA genes, Jung et al., 2025), we could provisionally assign our strains to the following species: *Lobosphaera incisa* strain LC2 (Figure 1j–l), *Pseudochlorella signiensis* (Figure 1g–i), and *Klebsormidium elegans* (two isolates: strain C12 and C14; Figure 1a–f).

The grit crust is characterized by a community dominated by lichen encrusting the surface and an interior of stones (<6 mm) in an area with poorly developed soil. In the Atacama Desert, about 90% of the total water deposition originates from fog the occurs during the night until noon. High air humidity exceeding 90% even on the soil level of the grit crust area was recorded at various sites in the National Park Pan de Azúcar on a daily basis (Jung et al., 2025). High irradiation maxima of more than 1500 μmol photons · m^−2^ · s^−1^ were often recorded during cloud‐free periods (Jung et al., 2020). Mean annual temperatures (MATs) typically range between 12 (air) and 15°C (ground level), including highly dynamic daily fluctuations for biocrusts heating up to 60°C (Jung et al., 2020).

The isolates were free‐living species in the grit crusts. The algae were kept on Bold Basal Medium (BBM) with three times nitrogen (3 N BBM; Bischoff & Bold, 1963) at 20°C under low‐light conditions (50 μmol photons · m^−2^ · s^−1^). All cultures were visually examined for fungal or bacterial contamination and, if necessary, treated with fungicide (40 μg · mL^−1^ Carbendazim) and repeatedly and carefully transferred until the cultures appeared under the light microscope as clean and clonal. Afterward, the clonal cultures were grown without fungicide for about 4 weeks under the conditions mentioned above prior to the ecophysiological experiments (pre‐acclimation).

### Light‐ and temperature‐dependent photosynthesis and respiration

The ecophysiological experiments include the evaluation of light‐ and temperature requirements of photosynthesis, which were carried out according to Prelle et al. (2019) with minor adjustments. Prior to the measurements, a two‐point calibration of the measuring system was performed at 0% and 100% oxygen concentrations. Each culture was supplied with fresh 3 N BBM 4 to 6 days before the actual measurements, to ensure a logarithmic growth phase. The measurements were conducted in four parallel replicates in 3.1‐mL cuvettes; the medium was enriched with NaCO_3_ (2 mM) to avoid carbon limitation during the experiment. Oxygen concentration in cuvettes was continuously measured with oxygen‐optodes using OXY‐4 mini (PreSens Precision Sensing GmbH, Regensburg, Germany).

For light‐dependent photosynthesis curves (P‐I curves), the temperature was kept constant at 20°C. The oxygen concentration was measured at 11 increasing photon flux density levels ranging from 0 to ~1400 μmol photons · m^−2^ · s^−1^ of photosynthetically active radiation (PAR), using a non‐invasive oxygen dipping probe (DP sensors PreSens Precision Sensing GmbH, Regensburg, Germany). Measurements consisted of a 30‐min respiration (dark) phase to avoid potential photodamage, followed by a 10‐min photosynthesis (light) phase for each light level.

For temperature‐dependent photosynthesis curves, the photon fluence density was kept constant at 485 μmol photons · m^−2^ · s^−1^ at the light phases. The starting temperature was 5°C, and the maximum was 40 with 5°C increments. Each temperature level consisted of a 30‐min dark phase and a 10‐min light phase. During the dark phase, the first 20 min were used to short‐term acclimatize the cultures, and the last 10 min were used to calculate respiration. This methodological approach was developed for *Klebsormidium* species isolated from high alpine biocrusts (Karsten & Holzinger, 2012). The light compensation point (*I*_c_) is defined as the photon fluence rate at which gross photosynthesis equals 0. The light saturation point was calculated (*I*_k_ = *P*_max_/alpha) as a convenient indicator of the transition between the light‐limited and light‐saturated regions of the P‐I curve.

After the experiment, the chlorophyll *a* concentration was measured and used as a reference parameter for photosynthesis and respiration. Therefore, each replicate culture was transferred to an individual glass fiber filter (Whatman, Dassel, Germany), which was extracted in 3 mL dimethylformamide (DMF) and incubated in the refrigerator, in the dark for at least 24 h. After incubation, the samples were centrifuged (Heraeus Megafuge 16R) at 700 x *g* for 10 min. The reaction vessels were darkened with an aluminum foil cover to minimize any degradation of chlorophyll *a* due to exposure to light. The absorbance of the supernatant was then measured between 350 and 750 nm in a spectrophotometer (Lambda 2, Perkin Elmer, United States). The chlorophyll *a* concentration was calculated according to the following formula (Porra et al., 1989):

$$\begin{array}{c} \mathrm{Chl}\;a\;\left[g\;\cdot \;m^{-3}\right]=12\left(E664-E750\right)-3.11\left(E647-E750\right) \end{array}$$

### Desiccation tolerance

The experiment followed the procedure described by Karsten et al. (2016). Two hundred fifty microliters of cell culture were transferred to a glass fiber filter (25 mm diameter, Whatman) and placed on a table in a desiccation chamber filled with activated silica beads, which resulted in a relative humidity of 10.9% ± 0.5%. The relative humidity in the chambers was continuously recorded using a multifunctional data logger (MSR 145 W; MSR Electronics GmbH, Switzerland). The experiments were done in triplicate at 20°C. The photosynthetic potential of each culture was followed over time using a chlorophyll fluorometer (PAM 2500, Heinz Walz, Effeltrich, Germany); variables were adjusted to ensure a *F*_t_ value of >0.1. We chose the effective photochemical quantum yield of photosystem II, Y(II), as an indicator of cell viability and the critical point of dehydration because it provides a sensitive and non‐invasive measure of photosynthetic performance, directly reflecting the functional integrity of the photosynthetic apparatus under stress conditions. A Y(II) value of 1 would theoretically indicate that all absorbed light energy was efficiently transferred to photosystem II for conversion into chemical energy (theoretical maximum, cannot be reached in vivo). Decreasing values reflect progressive impairment of photosystem II activity. The Y(II) values were recorded every 30 min; after Y(II) reached 0 during the desiccation process, the filters were rewetted with 250 μL culture medium and transferred to a water‐saturated chamber. To assess the recovery dynamics, Y(II) values were recorded 2 and 20 h after rewetting of the previously desiccated alga.

### Statistical analysis

All statistical analyses were done in R, version 4.2.1 (R Development Core Team, 2022) or Microsoft Excel. Photosynthetic‐irradiance curves were fit using the Walsby model in Excel, based on least‐square methods (Walsby, 1997). Temperature curves were fit using the Yan and Hunt (1999) model in R, also based on the least‐square model (Yan & Hunt, 1999).

## RESULTS

### Light‐dependent photosynthesis (P‐I curve)

**FIGURE 2:**
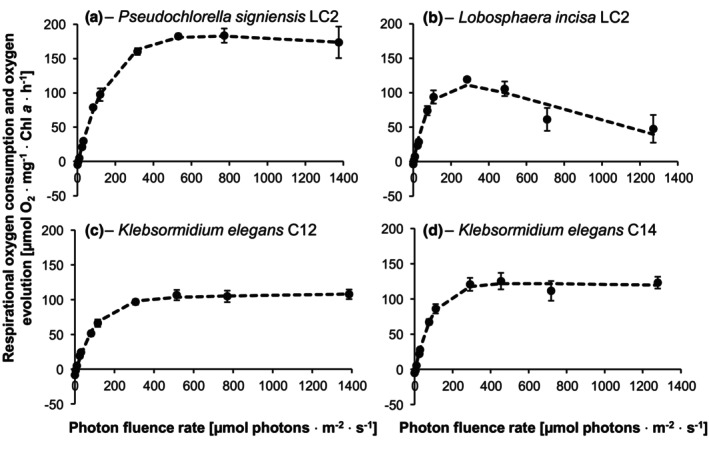
Photosynthetic‐irradiance curve of four microalgal cultures isolated from grit crusts in the Atacama Desert. The points represent the mean of the measured values (*n* = 4 ± standard deviation), the line represents the fitting curve after Walsby (1997). (a) *Pseudochlorella signiensis* C11, (b) *Lobosphaera incisa* LC2, (c) *Klebsormidium elegans* C12, (d) *Klebsormidium elegans* C14.

The photosynthesis‐irradiance curves revealed significant differences in photosynthetic performance among the algal strains (Figure 2). *Pseudochlorella signiensis* C11 exhibited the highest maximum photosynthetic rate (*P*_max_) at 183 μmol O_2_ · mg^−1^ Chl *a* · h^−1^, representing a 1.8‐fold increase compared to *Klebsormidium elegans* C12 (102 μmol O_2_ · mg^−1^ Chl *a* · h^−1^, Table 1, Figure 2e). This suggests a greater capacity for carbon fixation in *P. signiensis* C11 compared with the other investigated algae under high light conditions. The light utilization coefficient (alpha) was highest in *Lobosphaera incisa* LC2 (1.7 μmol O_2_ · mg^−1^ Chl *a* · h^−1^ / μmol photons · m^−2^ · s^−1^), indicating efficient photosynthesis at low‐light levels, consistent with its potential niche within shaded microhabitats of the grit crust. Notably, *L. incisa* LC2 reached light saturation (*l*_k_ = 68 μmol photons · m^−2^ · s^−1^) at a lower photon fluence rate than *K. elegans* C12 did (*l*_k_ = 99 μmol photons · m^−2^ · s^−1^), suggesting a lower light requirement for maximal photosynthetic performance. The light compensation point (*l*_C_) varied considerably, with *L. incisa* LC2 reaching compensation at only 2.3 μmol photons · m^−2^ · s^−1^, whereas *K*. *elegans* C12 required 7.8 μmol photons · m^−2^ · s^−1^.

**TABLE 1:** Mean values of the characteristic PI curve variables of four microalgae cultures isolated from grit crust of the Atacama Desert.

	*P*_max_	Alpha	β	*R*	*I*_k_	*I*_c_
*Pseudochlorella signiensis* C11	183.14	1.24	−0.02	−4.69	151.56	3.89
*Lobosphaera incisa* LC2	111.33	1.68	−0.08	−3.65	68.37	2.31
*Klebsormidium elegans* C12	101.82	1.11	0.00	−8.43	98.90	7.83
*Klebsormidium elegans* C14	121.88	1.40	0.00	−5.40	91.09	3.96

*Note*: *P*_max_—maximum photosynthesis in μmol O_2_ · mg^−1^ Chl *a* · h^−1^, *R*—respiration in μmol O_2_ · mg^−1^ Chl *a* h^−1^, alpha—light utilization coefficient in μmol O_2_ · mg^−1^ Chl *a* · h^−1^ / μmol photons · m^−2^ · s^−1^, β—photoinhibition coefficient in μmol O_2_ · mg^−1^ Chl *a* · h^−1^ / μmol photons · m^−2^ · s^−1^, *l*_k_—saturation PFD in μmol photons · m^−2^ · s^−1^, *l*_c_—light compensation point in μmol photons · m^−2^ · s^−1^.

Photoinhibition (β), representing a decline in photosynthetic efficiency under excessive light, was minimal in most strains. However, *L. incisa* LC2 exhibited a significant degree of photoinhibition (−0.08 μmol O_2_ · mg^−1^ Chl *a* · h^−1^ / μmol photons · m^−2^ · s^−1^), suggesting a greater sensitivity to excess light energy. In contrast, both *Klebsormidium* strains (C12 and C14) showed no evidence of photoinhibition, indicating robust photoprotective mechanisms.

Respiration among all investigated species varied between −3.7 and −8.4 μmol O_2_ · mg^−1^ Chl *a* · h^−1^ (Table 1), which was much lower than the light‐saturated photosynthetic rates. This result suggests that respiratory losses accounted for only a small fraction of the total photosynthetic capacity.

### Temperature‐dependent photosynthesis and respiration activity

**FIGURE 3:**
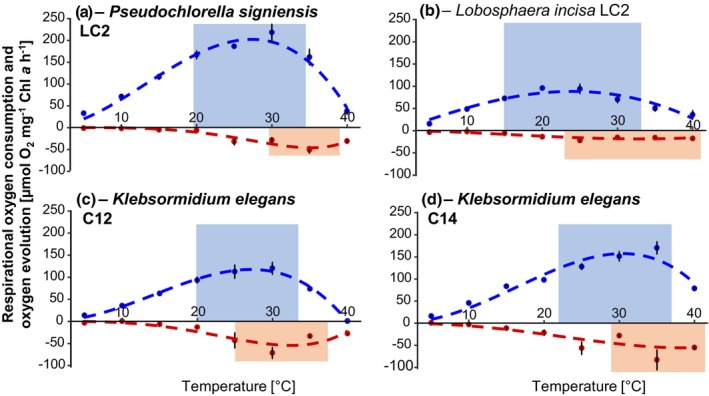
Temperature‐dependent oxygen production in light conditions (blue) or oxygen consumption in dark phases (red) of four microalgal cultures isolated from grit crusts in the Atacama Desert. The points represent the mean of the measured values (*n* = 4 ± standard deviation), the dotted line is the fitting curve after Yan and Hunt (1999), and the boxes represent the optimum range, in which 80% of the maximum photosynthesis (light blue box) or respiration (orange box) would be reached based on the fitting curve. (a) *Pseudochlorella signiensis* C11, (b) *Lobosphaera incisa* LC2, (c) *Klebsormidium elegans* C12, (d) *Klebsormidium elegans* C14.

Temperature‐dependent photosynthesis revealed that the highest oxygen production was strain dependent between 20 and 35°C (Figure 3). *Pseudochlorella signiensis* C11 exhibited the highest oxygen production at 27.6°C (210 μmol O_2_ · mg^−1^ Chl *a* · h^−1^), but its optimal temperature range (19.7–34.2°C) was the narrowest observed. *Lobosphaera incisa* LC2 displayed the broadest optimal temperature range (14.6–32.8°C), suggesting greater physiological flexibility (Figure 3, Table 2).

**TABLE 2:** Mean values of the temperature‐dependent photosynthesis and respiration curves of four microalgae cultures isolated from grit crust of the Atacama Desert.

	Photosynthesis				Respiration			
	*P*_max_	*T*_opt_	*T*_max_	*T*‐80%	*R*_max_	*T*_opt_	*T*_max_	*T*‐80%
*Pseudochlorella signiensis* C11	209.5	27.6	41.3	19.7 to 34.2 (Δ = 14.5)	−46.5	35.2	43	29.8 to 39.1 (Δ = 9.3)
*Lobosphaera incisa* LC2	88.4	24	43.1	14.6 to 32.8 (Δ = 18.2)	−18.6	33	50	23.2 to 41.1 (Δ = 17.9)
*Klebsormidium elegans* C12	117.4	27.2	40	19.7 to 33.3 (Δ = 13.6)	−54.5	32.5	41.9	26.4 to 37.2 (Δ = 10.8)
*Klebsormidium elegans* C14	157.1	30.5	44.4	22.2 to 37.2 (Δ = 15)	−66	36	46.9	29 to 41.4 (Δ = 12.4)

*Note*: *P*_max_—maximum photosynthesis in μmol O_2_ · mg^−1^ Chl *a* · h^−1^, *R*_max_—maximum respiration in μmol O_2_ · mg^−1^ Chl *a* · h^−1^, *T*_opt_—optimum temperature for photosynthesis or respiration, respectively, in°C, *T*_max_—maximum temperature for photosynthesis or respiration, respectively, in°C, *T*‐80%—range, in which at least 80% of maximum photosynthesis or respiration could be measured in°C.

Respiration rates were generally lower than photosynthetic rates, with optimal temperatures for respiration consistently 5–10°C higher than those for photosynthesis. *Klebsormidium elegans* C14 exhibited the highest respiration rate (−66 μmol O_2_ · mg^−1^ Chl *a* · h^−1^), while *L. incisa* LC2 had the lowest (−18.6 μmol O_2_ · mg^−1^ Chl *a* · h^−1^). The narrow optimal temperature range for respiration in *K. elegans* C14 (29.8–39.1°C) suggests a limited capacity to maintain metabolic activity outside of this range.

### Desiccation tolerance experiment

**FIGURE 4:**
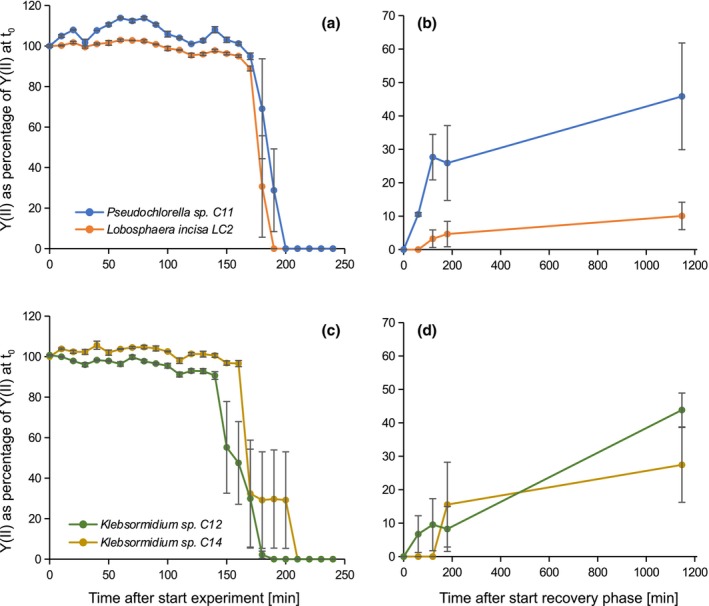
Effect of controlled desiccation (a, c) and rehydration (b, d) on the effective quantum yield (Y(II)) of PSII to four microalgal cultures isolated from biocrusts (*n* = 3 ± standard error); for better visuality, only two strains were presented in each panel (a, b) *Pseudochlorella signiensis* C11, *Lobosphaera incisa* LC2; (c, d) *Klebsormidium elegans* C12 + C14). Effective quantum yield values were standardized to the starting Y(II) to 100% for better comparison.

The initial values of the effective Y(II) were between 0.53 and 0.63, which indicates viable microalgal cultures. The desiccation tolerance experiment (Figure 4) demonstrated a rapid decline in Y(II) in all cultures upon exposure to very low relative air humidity (10%–15%). After approximately 230 min, Y(II) reached 0 in all strains. Following rehydration, *Pseudochlorella signiensis* C11 exhibited the highest recovery of PSII activity, reaching 49% of its initial value after 20 h. Both *Klebsormidium* strains showed similar but lower recovery rates (ranging from 37% to 39%), whereas *Lobosphaera incisa* LC2 exhibited the lowest recovery (11%).

## DISCUSSION

This investigation provides a comparative ecophysiological assessment of typical green algae isolated from a recently discovered biocrust type, grit crust, which dominates the coastal range of the hyperarid Atacama Desert, Chile. Our results revealed significant interspecific variation in photosynthetic performance, temperature tolerance, and desiccation recovery, highlighting physiological plasticity and flexibility that enable algal survival in this extreme environment. In addition, our study contributes to a growing, but still limited, understanding of terrestrial algal ecology in South America, a region historically underrepresented in phycological research.

### Light‐dependent photosynthetic and respiratory activity (P‐I curve)

The observed differences in P‐I curves provide insights into the light requirements and acclimation strategies of grit crust algae. All the strains were characterized by moderate to high *P*_max_ values, indicating potentially high photosynthesis activity under favorable conditions. Previous studies on *Klebsormidium* strains isolated from Tyrolean alpine biocrusts measured only half of the *P*_max_ rates (46–80 μmol O_2_ · mg^−1^ Chl *a* · h^−1^, Karsten et al., 2013) of the strains in this study, but the values were similar to those of *Klebsormidium* isolates from South African drylands (Karsten et al., 2016). This comparison supports the notion that photosynthetic capacity is tightly linked to habitat‐specific light regimes, with Atacama strains outperforming their alpine counterparts, likely as a result of chronic exposure to high irradiance and limited precipitation input rather than atmospheric dryness. The ability to maintain photosynthetic function under fluctuating hydration conditions may thus reflect an adaptive balance between efficient light utilization and desiccation tolerance. A further aspect to consider is that photosynthetic activity in both free‐living algae and lichens requires the presence of liquid water. In the coastal Atacama, hydration occurs almost exclusively during the night, when fog droplets no longer evaporate due to ground heat release but instead wet the organisms and the grit surface. Previous in situ measurements clearly showed that even during dense daytime fog, no photosynthetic activity took place (Jung et al., 2020). The activity window extends from the early morning until late morning (typically 11:00–12:00 h), when increasing irradiance and declining surface moisture rapidly inhibit further activity. Consequently, although irradiance may already be high toward midday, effective photosynthesis is restricted to the period of sufficient surface moisture during the morning hours. Such physiological flexibility in water‐use and recovery dynamics appears to be a key factor shaping the ecological success of green algae in hyperarid grit crust environments.

The *I*_C_ values for the strains in this study were below 10 μmol photons · m^−2^ · s^−1^, which suggests low‐light acclimation (Sonnewald, 2014). This is particularly noteworthy given the high irradiance levels characteristic of the Atacama Desert (up to 1500 μmol photons · m^−2^ · s^−1^). Although the Atacama Desert lacks shaded habitats on the macroscale, it is crucial to consider the microhabitat context. Grit crusts are not uniformly exposed to high insolation. Whereas most lichen grows on the stone's surface, the free‐living algae preferentially grow hypolithically underneath quartz and granitoid stones (Jung et al., 2024). Natural quartz pebbles transmit only a small fraction of incident light, but values vary greatly with clarity and thickness; for ~6 mm natural quartz pebbles a conservative estimate is ~5%–30% of incident visible light (Bosch et al., 2024). Consequently, the undersides of these stones provide shaded microhabitats, and hence, algal cells experience reduced light penetration. Thus, the low *I*_C_ may reflect acclimation to these low‐light niches within the grit crust. This assumption is supported by similar analyses that were conducted on the intact grit crust communities and not on isolated strains, for which *l*_C_ was around 460 μmol photons · m^−2^ · s^−1^ (Jung et al., 2020). This significantly higher *l*_C_ can be explained by the dominance of microlichens with Trebouxioid green algae growing on the surface of the grits because lichens in general have greater photosynthetic performances than isolated or free‐living green algae due to their symbiotic fungus (Candotto Carniel et al., 2015). Although photobionts in general experience photoprotection by the fungal host, free‐living green algae rely on shaded microhabitats such as underneath stones or in deeper layers of biocrusts. Recent data on the microbiome also showed that free‐living green algae only play a minor role within the grit crust community compared to lichenized green algae (Jung et al., 2024), which might indicate that the abiotic factors of the environment are pushing the ecophysiological capacity of the algae to their limits. In addition, an ecologically convincing explanation for low‐light acclimation in microalgae under high natural insolation was given by Gray et al. (2007), who investigated biocrusts of North American deserts. In the communities studied by Gray et al. (2007), abundant green microalgae vertically occupied microenvironments within the biocrust, which resulted in massive self‐shading and, thus, photoprotection. The observed differential susceptibility of individual algal taxa points to a complex spatial arrangement of the algal species in such a microenvironment, which seems to be structured in response to the vertical attenuation of solar light (Gray et al., 2007). In addition, morphology and chloroplast architecture likely modulate photosynthetic performance in interaction with external light conditions. The filamentous organization of *Klebsormidium* strains may increase light scattering and self‐shading, thereby generating microenvironments with reduced irradiance. Moreover, strain‐specific capacities for photoprotective responses—such as pigment adjustments, carotenoid accumulation, non‐photochemical quenching, and chloroplast rearrangement—reflect genetically fixed but environmentally modulated traits that together determine photosynthetic plasticity. The resulting microstructure contributes to a high degree of self‐shading and, thus, photoprotection of individual filaments (Karsten et al., 2010). These examples highlight the importance of considering microscale environmental variation when interpreting physiological traits. We cannot, however, exclude the acclimation to the artificial culture conditions, even if they were without desiccation stress and low light. Nevertheless, the strains from the grit crusts were only in culture for 1 year with very low growth, which speaks against culture artifacts.

Low‐light adaptation in most algae is typically linked to strong photoinhibition under excessive solar radiation. The underlying reasons are that algae acclimated to low‐light environments develop extremely sensitive photosynthetic systems optimized to capture minimal photon flux, often through modifications in light‐harvesting proteins and pigments. However, these highly efficient, low‐light‐adapted systems become particularly susceptible to photodamage when suddenly exposed to high solar radiation (Häder et al., 2002). In contrast to this statement, three out of four strains in this study lacked strong photoinhibition, suggesting robust photoprotective mechanisms. These mechanisms could involve efficient non‐photochemical quenching (NPQ) or the accumulation of protective pigments like carotenoids (Bonente et al., 2008). The rare observation of photoinhibition in this study aligned with findings on *Klebsormidium*, *Interfilum*, and *Chlorella ohadii* from other biocrusts (Donner, Glaser, et al., 2017; Karsten et al., 2010, 2013, 2014, 2016; Levin et al., 2021); *Trentepohlia* from stony surface (Holzinger et al., 2023); and *Diplosphaera chodatii* from a tree bark (Medwed et al., 2021), which also did not show photoinhibition under high light conditions. Taken together, the consistent absence of photoinhibition across various biocrust and terrestrial algal taxa (Chlorophyta and Streptophyta) underscores a remarkable physiological plasticity and tolerance to irradiance fluctuations typical for exposed soil environments. Confirmed by the many other studies mentioned above, the data presented here clearly point to a generally high photo‐physiological plasticity of biocrust microalgae, which explains their remarkable tolerance against diurnally and seasonally fluctuating light conditions. The assumed vertical light gradients in grit crusts and their effect on microalgae distribution on the microscale require further investigation by microsensor studies to explore in detail the physical–chemical microenvironment and by confocal laser scanning microscopy to localize the precise position of individual algal species within the grit crust, for example.

### Temperature‐dependent photosynthetic and respiratory activity

The temperature‐dependent photosynthesis experiments revealed a relatively narrow optimal temperature for all strains, ranging from 24 to 30.5°C. These values were generally lower or similar to reports from other *Klebsormidium* isolates from African biocrusts (30–35°C, Karsten et al., 2016). The terrestrial *Chlorella vulgaris* (closely related to *Lobosphaera incisa* and *Pseudochlorella signiensis*) exhibited higher optima (~35°C; Aigner et al., 2020). The lower optimum temperatures for the strains might seem surprising at first, given that Atacama is the driest nonpolar desert in the world; nevertheless, it is considered a cold climate desert according to the Köppen climate classification. The grit crusts were collected in the coastal region, which is heavily influenced by coastal fog. The foggiest season is from July to September (spring) with temperatures between 20 and 25°C at noon (Sotomayor & Drezner, 2019). Also, in summer, the temperatures rarely exceed 30°C close to the coast (Cáceres et al., 2007), with moderate day–night temperature differences (Δ 10°C). The strains were characterized by a rather broad temperature range for optimal photosynthesis (Δ 15–18°C). Similarly, the Arctic *K. subtilissimum* exhibited a broad optimum range (Δ 20°C; Karsten et al., 2014). In contrast, *Klebsormidium* isolated from South Africa had a small optimum range (Δ 10°C; Karsten et al., 2016).

At temperatures between 10 and 15°C, respiration rates were too low to be measured reliably with our setup, which was in contrast to photosynthesis, which produced measurable oxygen increases at 5°C. This implies that, especially at low temperatures, there is a positive net carbon gain, which decreases with increasing temperature. The respiration also decreases in most of the strains at high temperatures, preventing a negative carbon gain at 40°C. The optimal temperature for respiration was consistently 5–10°C higher than for photosynthesis. This uncoupled relationship between temperature effects on photosynthesis and respiration has been reported several times for other terrestrial green algae, including *Interfilum* and *Klebsormidium* species from biocrusts and unicellular green algae (*Chlorella vulgaris*) from soil (Aigner et al., 2020; Karsten et al., 2014; Karsten & Holzinger, 2012), as well as from benthic diatoms and mosses (Hancke & Glud, 2004; Perera‐Castro & Nadal, 2025). A reasonable explanation for these conspicuously different temperature requirements of both physiological processes is that photosynthesis is more strongly influenced by irradiance than by temperature, whereas respiration depends on temperature‐sensitive enzymatic reactions (Atkin & Tjoelker, 2003; Karsten et al., 2014). Photosynthesis is primarily controlled by light‐related physicochemical processes such as light absorption, energy transfer, etc., all closely associated with thylakoids. In contrast, respiration is a biochemically complex pathway consisting of numerous enzymes, which are localized in different cellular compartments and that exhibit different temperature optima (Atkin & Tjoelker, 2003). Consequently, the most temperature‐sensitive enzyme of respiration acts as a bottleneck and would control the complete process.

### Desiccation‐dependent activity of photosystem II

The desiccation tolerance experiment yielded the most nuanced results. The partial recovery of photosynthetic activity following rehydration suggests that these algae possess some degree of desiccation tolerance, but it is not absolute. The relatively low recovery rates of <50% of starting values observed in most strains from the Atacama Desert were unexpected. In contrast, *Klebsormidium* strains (E‐Clade) from biocrusts in semiarid regions (United States) exhibited higher recovery values of 70% to 95% based on the same method (Donner, Ryšánek, et al., 2017). A comparison with *Klebsormidium* cultures from Central European biocrusts (B‐ and E‐Clades) revealed high recovery rates (above 60%) after rehydration for *K. dissectum* and *K. subtile*, and similar recovery rates for *K. nitens* and *K. flaccidum* (Donner, Glaser, et al., 2017). Similarly, a study from South African *Klebsormidium* strains (G‐clade) also reported some strains with nearly full recovery, whereas other phylogenetically close strains were characterized by rather low recovery rates similar to our study (Karsten et al., 2016).

Concerning *Klebsormidium*, Mikhailyuk et al. (2015) suggested an interesting concept based on correlation analysis between molecular phylogeny (clade) and desiccation tolerance connected with morphological traits. G‐clade and F‐Clade *Klebsormidium* species are considered xerophytic because of their morphological traits, that is, their thick cell walls and narrow cells, strongly curved filaments arranged in ball‐like aggregations, and cluster‐like colonies. These morphological features prevent, or at least delay, water loss from the cell. In contrast, the *Klebsormidium* E‐Clade, to which strains C12 and C14 belong, is characterized by a delicate cell wall compared to the G‐Clade. Further, most members of this clade tend to disintegrate more rapidly (Mikhailyuk et al., 2015). This trait would allow more rapid vegetative reproduction but limit the self‐protecting potential of the filaments. A key observation concerning desiccation tolerance in *K*. *crenulatum* (F‐clade) is related to unique mechanical properties of cross‐cell walls, which are highly flexible during water loss, as they strongly undulate during cell shrinkage without disturbing the remaining cell (Holzinger & Karsten, 2013). The atypical *Klebsormidium* cell wall contains mainly callose and, hence, is poor in otherwise typical cellulose, which explains its outstanding flexibility upon desiccation and shrinkage. This strategy might not be limited to members of the F‐Clade and could theoretically be present in other *Klebsormidium* clades as well.

Further comparisons can be drawn with the terrestrial *Diplosphaera chodatii*, a unicellular green alga from the same class as *Pseudochlorella signiensis* C11 and *Lobosphaera incisa* LC2. Following rehydration, *D. chodatii* exhibited a recovery rate of 85% (Medwed et al., 2021), significantly exceeding the values observed for *L*. *incisa* LC2 (11%) and *P. signiensis* C11 (49%) in our study. The reasons for this striking difference might be that *D. chodatii* was isolated from the bark of a solitary tree, that is, a completely different habitat. The bark is a challenging environment because the associated biofilm is usually very thin, with few cell layers and consists mainly of one algal species. Thus, *D. chodatii* on a tree bark has only minimal physical protection by the biofilm matrix against desiccation, and hence physiological and biochemical acclimation processes, like acclimation of low molecular weight carbohydrates, are essential to cope with this stressor (Medwed et al., 2021).

Several factors could explain the rather low recovery rates of the grit crust algae from the Atacama Desert. First, the method used to assess desiccation tolerance (PAM fluorometry) measures the recovery of PSII activity, which is only one aspect of desiccation tolerance. Other cellular processes, such as membrane integrity and DNA protection, may also be compromised during dehydration and might contribute to the limited recovery observed. Second, the specific desiccation protocol (relative air humidity of ~11%) might have been more severe than the algae typically experience in their natural habitat. The frequent fog events in the Atacama Desert may provide sufficient moisture to prevent complete desiccation, selecting for algae that can tolerate partial dehydration rather than prolonged, extreme dryness (Lehnert et al., 2018). In contrast to inland Atacama, the relative air humidity at the coast drops rarely below 20% and is on average around 40%–80% (Cáceres et al., 2007).

## CONCLUSIONS

This study provides insight into the ecophysiological performance of free‐living algae in the grit crust of the Atacama Desert. The algal strains from different phylogenetic positions showed low‐light adaptation, and most of the strains managed high radiation without indication of photoinhibition. The low‐light adaptation most likely reflects ecological microhabitat conditions of the grit crusts, where algae are often found beneath quartz stones. All strains were desiccation‐tolerant but recovered less well from the stress compared to other studies using the same approach. This may reflect the particular conditions at the sampling site. Sampling took place close to the coast, with regular fog events that led to moderate air humidity, although precipitation in the Atacama Desert is low.

## AUTHOR CONTRIBUTIONS

**K. Glaser:** Conceptualization (equal); data curation (equal); writing – original draft (lead). **F. Kuschel:** Data curation (equal); investigation (lead); validation (equal); writing – review and editing (supporting). **L. R. Prelle:** Data curation (equal); supervision (equal); writing – review and editing (equal). **P. Jung:** Data curation (equal); funding acquisition (equal); resources (equal); writing – review and editing (equal). **U. Karsten:** Funding acquisition (equal); supervision (equal); writing – review and editing (equal).

## FUNDING INFORMATION

PJ was funded by the German Research Council [Grit Life JU 3228/1‐1] and the Carl‐Zeiss Foundation [P2023‐03‐051]. UK thanks the German Science Foundation (DFG) for funding the project CRUSTWEATHERING (KA899/32‐1) in the frame of the priority research program SPP 1803 “EarthShape: Earth Surface Shaping by Biota”.
